# Towards a Dynamic Clamp for Neurochemical Modalities

**DOI:** 10.3390/s150510465

**Published:** 2015-05-04

**Authors:** Catalina Maria Rivera, Hyuck-Jin Kwon, Ali Hashmi, Gan Yu, Jiheng Zhao, Jianlong Gao, Jie Xu, Wei Xue, Alexander G. Dimitrov

**Affiliations:** 1 Departments of Mathematics, Washington State University Vancouver, Vancouver , WA 98686, USA; E-Mail: catalina.maria.rivera@emory.edu; 2 Department of Physics, Emory University, Atlanta, GA 30332, USA; 3 Department of Electrical and Computer Engineering, McMaster University, Hamilton, ON L8S4L8, Canada; E-Mail: hyuck.jin.kwon0@gmail.com; 4 Department of Bioengineering, Stanford University, Stanford, CA 94305, USA; E-Mail: alihashmi_87@hotmail.com; 5 Departments of Mechanical Engineering, Washington State University Vancouver, Vancouver, WA 98686, USA; E-Mails: yugan0000@gmail.com (G.Y.); jiheng.zhao@wsu.edu (J.Z.); gaojl1015@gmail.com (J.G.); 6 Department of Mechanical and Industrial Engineering, University of Illinois at Chicago, Chicago, IL 60607, USA; E-Mail: jiexu@uic.edu; 7 Department of Mechanical Engineering, Rowan University, Glassboro, NJ 08028, USA; E-Mail: xuew@rowan.edu

**Keywords:** carbon nanotube sensors, microfluidics, neural modeling, dynamic clamp

## Abstract

The classic dynamic clamp technique uses a real-time electrical interface between living cells and neural simulations in order to investigate hypotheses about neural function and structure. One of the acknowledged drawbacks of that technique is the limited control of the cells' chemical microenvironment. In this manuscript, we use a novel combination of nanosensor and microfluidic technology and microfluidic and neural simulations to add sensing and control of chemical concentrations to the dynamic clamp technique. Specifically, we use a microfluidic lab-on-a-chip to generate distinct chemical concentration gradients (ions or neuromodulators), to register the concentrations with embedded nanosensors and use the processed signals as an input to simulations of a neural cell. The ultimate goal of this project is to close the loop and provide sensor signals to the microfluidic lab-on-a-chip to mimic the interaction of the simulated cell with other cells in its chemical environment.

## 1. Introduction

**Figure 1:**
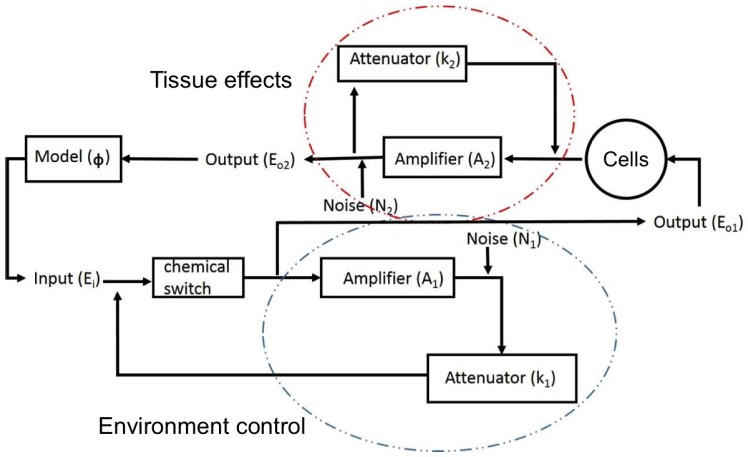
Proposed dynamic clamp feedback loop for neurochemical modulation. A chemical input is supplied to a group of cells, and their chemical response is fed to a mathematical model, which, in turn, simulates the release of chemicals by the modeled neuron. Those are instantiated by the microfluidic system through feedback environment control and affect the input chemical concentration of the environment akin to the effects produced by a live neuron.

The original dynamic clamp technique uses a real-time electrical interface between living cells and neural simulations in order to investigate hypotheses about neural function and structure [1,2]. It has been applied successfully to dissect the functionality of single neurons and small neural circuits. However, it has two major drawbacks [3]: the electrodes can clamp electrically only a small section of a cell and, hence, frequently do not control the electrical state of whole cells; and all control to-date has been concentrated on the electric properties of neurons, neglecting their chemical state. As noted in [3], the latter has been done by necessity, since until recently, registering or controlling the chemical state of either neurons or the chemical environment in which they reside has been essentially impossible with the level of precision needed to simulate the appropriate dynamics of the various extracellular chemical players. In this manuscript, we present an analogue to the dynamic clamp method, which includes the simulation and control of the effects of chemicals associated with neural signaling. To achieve that, we use the emergent discipline of microfluidics [4,5], which deals specifically with the behavior, manipulation and precise control of fluids at the microscale. The long-term goal of this project is a device as outlined in Figure 1, in which cell cultures or slices (cells) co-exist and interact with neural models. We report the initial steps and results on this project.

Our complex dynamic clamp system is enabled by innovations in microfluidics and sensor technology, especially by embedded, on-chip sensors. Advances in lab-on-a-chip microfluidic systems have demonstrated great potential to revolutionize biomedical research. Novel devices have been developed for drug delivery [6], neuron manipulation [7–9] and immune assays [10,11]. As more functions are integrated into one stand-alone lab-on-a-chip system, each component needs to be miniaturized while maintaining the same performance. As a result, nanosensors made of nanomaterials have become an ideal candidate. Single-walled carbon nanotubes (SWCNTs) are well known for their outstanding electrical, mechanical and chemical properties. They are particularly suitable for biosensing applications due to their large surface areas and high electrical conductivity. Their tubular structure provides a large number of sites for aqueous or gaseous molecules to absorb and react. The absorption of molecules alters the electrical property of the nanotube, resulting in a detectable resistance change. This phenomenon enables label-free detection, and the required measurement system is typically simpler and less expensive. On the other hand, many microfluidic devices have been developed to precisely stimulate neurons on a chip, and most of them use well-patterned electrodes [12,13]. In terms of chemical stimulation, segmented co-culture chambers have been used to separate fluids, so that specific chemical cues can be applied to regions of interest [14,15]. However, to our knowledge, micro-spatiotemporal variations in chemical cues have yet to be exploited for neuroscience applications.

In this manuscript, we present the results of experiments and simulations of fluid flow with changing Ca^2+^ concentrations and their effects on simulations of neural cells with Ca^2+^-selective channels. Ca^2+^ is a universal signaling molecule in the nervous system [16,17]. Typically, it is considered a vital intracellular second messenger. However, the existence of cell-surface detectors for extracellular Ca^2+^ indicate that it functions as an extracellular signaling molecule, as well. According to [18], cells become major sources and sinks for Ca^2+^ during cytoplasmic Ca^2+^ signaling events, because of the activation of Ca^2+^ export (e.g., by the plasma membrane Ca^2+^, ATPase, PMCA) and by Ca^2+^ entry (e.g., by store-operated channels). Although these processes ultimately balance, transient spatiotemporal gradients of extracellular Ca^2+^ can be generated during normal operation. In addition to its physiological significance, we chose to use Ca^2+^ because it is also easy to handle in the microfluidic devices (as water-soluble CaCl_2_). Furthermore, the dissociation of Ca^2+^ in water provided an ionic solution, which could be registered with the currently available set of sensors in the microfluidic lab-on-a-chip. However, the techniques developed in this manuscript can be used with most other neuromodulators, allowing for the recreation of the natural dynamics of the extracellular medium, which so far has been inaccessible. We instantiate some of the chemical simulations on chip and present the response of the neural simulation to the measured Ca^2+^ concentration. Currently, we have not yet closed the loop, in which the neural simulation will drive the control signals that determine the extracellular concentration of neuromodulators (like Ca^2+^ here).

## 2. Experimental Section

### 2.1. Microfluidic Lab-on-a-Chip

Microfluidics is an emerging technology that deals with the behavior, precise control and manipulation of fluids at the microscale [4,5]. Microfluidics technology enables the novel concept of lab-on-a-chip that aims at integrating one or more functions on a single chip of a few centimeters in size for chemical and biological experiments [19–22].

#### 2.1.1. Lab-on-a-Chip Design

**Figure 2:**
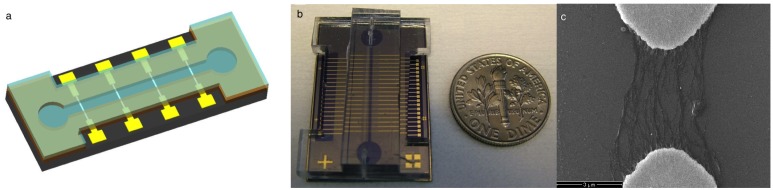
Schematic view of the microfluidic device used to perform the experiments. (**a**) Schematic of the designed lab-on-a-chip; (**b**) an implementation of the lab, with a dime for size comparison; (**c**) aligned single-walled carbon nanotubes (SWCNTs) as integrated nanosensors.

The main experimental system consists of the microfluidic chip shown in Figure 2. Such a chip is able to generate various concentration configurations. The chip also contains an array of carbon nanotube-based nanosensors that can respond selectively to changes in concentrations of ions. The proposed use of this chip is to introduce single ionic species through the corresponding channels and to register their concentrations with the on-chip nanosensors.

The microfluidic device is based on our recently-reported lab-on-a-chip system, which is fabricated with standard lithography and bonding methods [23,24]. In the fabrication process, a silicon wafer serves as the substrate and is cut into 2 cm × 3 cm pieces with a dicing saw machine. Multi-teeth electrodes with a 5 μm gap are used to ensure the controlled dielectrophoresis (DEP) alignment of nanosensors. The electrodes are fabricated by patterning metal layers of Cr and Au on the silicon wafer surface through metal sputtering, optical lithography and wet etching. Then, the negative photoresist SU-8 3050 is spin-coated onto the Si surface as the second layer, which contains all of the microfluidic components. The predesigned microchannels are transferred from the photomask to the SU-8 layer with UV lithography. The protected SU-8 layer under the photomask is dissolved in the SU-8 developer, leaving only the exposed, cross-linked SU-8 structure, which encloses the micro-channel network used in the device. The last step is bonding a piece of PDMS to SU-8 to form a sealed channel. Surface treatment is necessary in the bonding step [25]. Oxygen plasma and 5% 3-aminopropyltriethoxysilane (APTES) solution are used for the SU-8 surface treatment, which introduces amine (Si-NH_2_) groups onto the surface. For PDMS, oxygen plasma is used to generate silanol (Si-OH) groups. The covalent bonds of Si-O-Si formed by amine and silanol chemical groups bond the SU-8 and PDMS tightly.

The SWCNT nanosensors are integrated into the sealed microfluidic device after the SU-8-PDMS bonding. This integration is enabled by the in-channel dielectrophoresis process. A small volume of SWCNT dispersion is injected into the microchannel. An AC signal, with a frequency of 5 MHz and a peak-to-peak voltage of 10 V, is applied across a pair of electrodes to generate an electric field in the solution. This field induces electric dipole moments in dispersed SWCNTs and forces them to rotate along the electric field lines. The SWCNTs are eventually “captured” by the dielectrophoretic forces and land on the substrate. As the SWCNTs bridge the gap between the electrode pair, they become a part of the circuit and change the distribution of the electric field. This change creates a small potential drop of 0.5–2 V between the electrode pair, which can be visually observed on an oscilloscope.

The lab-on-a-chip system with predesigned microfluidic structures can precisely control the fluids in our system. The schematic structure of the microfluidic device is illustrated in Figure 2a, and the optical image of a fabricated device is shown in Figure 2b. Scanning electron microscopy (SEM) inspection shows that the SWCNTs are aligned between electrodes, as shown in Figure 2c. The SWCNTs are in the form of small bundles, and they are only observed near the electrode tips. This demonstrates that the dielectrophoresis process is an “active” approach to deposit SWCNTs, only placing them on locations with the strongest electric fields. This device, slightly larger than a dime, consists of an inlet, an outlet, a microfluidic channel and an electrode array with integrated nanosensors. It was fabricated to experimentally test the performance of the ion sensors, as described in next section. The new mixer also presented in this work will be attached to the device to create the desired conditions.

#### 2.1.2. Sensor Design

Calcium ion sensors have been investigated previously using various approaches. Among them, ion-selective electrodes have proven to be an effective method for Ca^2+^ detection [26,27]. Membranes inside these electrodes allow calcium ions to pass through for selective detection. In addition, such calcium ion sensors have been successfully integrated into microfluidic devices for real-time detection [27]. In this work, we use a similar configuration with SWCNTs as the integrated nanosensors in a microfluidic system [28].

#### 2.1.3. Flow Simulations

**Figure 3:**
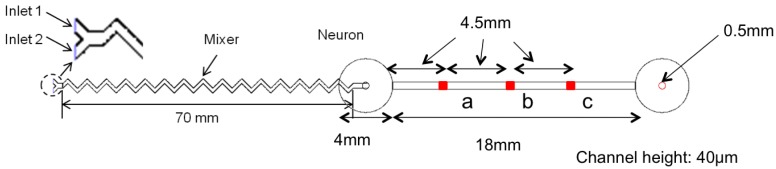
Schematic diagram of the designed mixer and neuron chamber.

**Figure 4:**
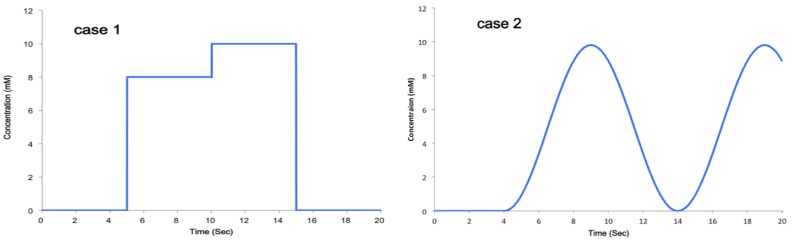
Concentration profile dynamics for two different cases. **Case 1:** combined step function, to illustrate the close transition between states; **Case 2:** sine wave, to test the device dynamics properties.

In order to predict the distribution of specific chemicals inside a microfluidic chip and to select appropriate geometries for the specific neurochemical dynamic clamp task, we used 3D CFD (computational fluid dynamics) techniques. In this study, the commercial software FLUENT^®^ 6.3 [29] was used to build the computational domain and the models for the microfluidic chip by using the finite element method (FEM). This method is suitable for simulating complex microfluidic flows, as demonstrated in our previous study [30]. Figure 3 shows the shape and dimensions of the microfluidic chip used in the experiment. The computational domain for the simulations consisted of 536,920 cells, 1,535,298 faces and 409,040 nodes. Navier–Stokes and diffusion-convection equations were employed to predict the flow and diffusion of the selected ion. The first-order schemes were used because they are known to provide better convergence of calculations than the second-order ones, although they provide less accurate results due to propagated error in numerical calculations. The SIMPLEC algorithm, as implemented in [29], was used for pressure-velocity coupling. An unsteady laminar flow model was selected since the concentration of the ion in the flow varied by time, and the maximum Reynolds number was less than 100. The flow in the microfluidic chip was assumed to be incompressible, non-isothermal and laminar for numerical calculation. Ca^2+^ was chosen as a target ion. Two cases of simulation were conducted by varying the concentration of the Ca^2+^ ion, as shown in Figure 4. The flow rate was fixed to 0.3 mL/min, the same as in the experimental conditions.

### 2.2. Neural Models

The Hodgkin–Huxley (HH) model is a mathematical model describing the behavior of a patch of a membrane neuron in which action potentials can be produced under certain conditions. The patch is considered mainly as a lipid bilayer, mostly impermeable, with certain proteins embedded in it considered as voltage-gated ion channels. This model is well understood and allows us to test the effects of the proposed novel engineering system without confounding them with novel effects in a neuronal model.

The system is modeled as a circuit where a capacitance *C_m_* represents the bilayer, and the non-linear conductances for Na^+^ and K^+^ represent the voltage-gated channels. There is also a constant “leak” conductance, for which *I_L_* = *g_L_*(*V* − *E_L_*) would correspond to the passive flow of ions through non-gated channels [31]. The conductances representing the Na^+^ and K^+^ voltage-dependent channels are *g_Na_* = *ḡ_Na_m*(*V*)^3^*h*(*V*) and *g_K_* = *ḡ_K_n*(*V*)^4^, where *ḡ_Na_* and *ḡ_K_* are the maximum values that the conductances can take. Therefore, by using certain gating variables *m, h, n*, which represent the fraction of a certain gate's type opened, it is possible to calculate the probability of a K^+^ channel being opened as proportional to *n*^4^, since this is composed of four identical gates and similarly for a Na^+^ channel as proportional to *m*^3^*h*, composed of four gates, three of them identical.

Each of the gating variables satisfies a first order differential equation:

(1) $$\frac{dn}{dt}=\alpha_{n}\left(V\right)\left(1-n\right)-\beta_{n}\left(V\right)n$$

(2) $$\frac{dm}{dt}=\alpha_{m}\left(V\right)\left(1-m\right)-\beta_{m}\left(V\right)m$$

(3) $$\frac{dh}{dt}=\alpha_{h}\left(V\right)\left(1-h\right)-\beta_{h}\left(V\right)h$$

where *α_i_*(*V*) is the rate of transition from closed to opened of gate type *i* ∈ {*n, m, h*}; conversely, β*_i_*(*V*) would be the rate of transition from opened to closed.

With all of this in mind, the equation for the circuit is:

(4) $$C_{m}\frac{dV}{dt}=g_{Na}\left(E_{Na}-V\right)+g_{K}\left(E_{K}-V\right)+g_{L}\left(E_{L}-V\right)+I_{\text{inj}}$$

#### 2.2.1. Calcium Channels

We focus our attention on a model in which, in addition to sodium and potassium channels, calcium channels are also considered [31]. The model would be described by the following system of equations, adding calcium channel dynamics to the standard HH model:

(5) $$C\frac{dV}{dt}=I_{o}-g_{L}\left(V-E_{L}\right)-I_{T}$$

(6) $$\frac{dh}{dt}=\frac{\left(h_{\infty }\left(V\right)-h\right)}{\tau_{h}\left(V\right)}$$

where:

(7) $$\begin{array}{c} I_{T}=m_{\infty }{\left(V\right)}^{2}hI_{\mathrm{cfe}}\left(V,{\left[Ca\right]}_{in},{\left[Ca\right]}_{\mathrm{out}}\right)\\ I_{\mathrm{cfe}}=P_{\text{max}}\frac{z^{2}F^{2}}{RT}V\left(\frac{{\left[Ca\right]}_{in}-{\left[Ca\right]}_{\mathrm{out}}e^{\frac{-\mathrm{zVF}}{RT}}}{1-e^{\frac{-\mathrm{zVF}}{RT}}}\right)\\ m_{\infty }\left(V\right)=\frac{1}{1+\exp\left(-\left(V+59\right)/6.2\right)}\\ h_{\infty }\left(V\right)=\frac{1}{1+\exp\left(\left(V+83\right)/4\right)}\\ \tau_{h}\left(V\right)=22.7+\frac{0.27}{\exp\left(\left(V+48\right)/4\right)}+\exp\left(-\left(V+407\right)/50\right) \end{array}$$

where *P_max_* is the permeability of the membrane, *R* is the ideal gas constant, *F* is Faraday's constant and *z* is the valence of the ion, which is 2 for the case of Ca^2+^. What sets the behavior of this model is the resting potential. In [31], the fixed point of the system is moved by changing *E_L_*. We see that for *E_L_* = −65 mV with fixed point (*V_rest_* = −61.01 mV, *h_rest_* = 0.0041), there is no action potential when a depolarizing current is applied. On the other hand, when *E_L_* = −80 mV with (*V_rest_* = −76.38 mV, *h_rest_* = 0.1606), a small depolarizing input induces an action potential.

Usually, it is assumed that the intracellular and extracellular concentrations are constant during the process. Here, however, we want to keep track of the Ca^2+^ concentration dynamics. First, we express the explicit dependence of the ionic current on concentration through *I_cfe_*. Next, we consider the dynamics of the intracellular concentration, by adding the following equation to the model:

(8) $$\frac{d\left[x\right]}{dt}=-\gamma I_{T}-\delta \left(\left[x\right]\right)$$

The first term is just a conversion from current to concentration flux, with *γ* = 0.002 μM·cm^2^/(ms·μA). This value is based on measurements done by [32] in cortical pyramidal neurons. The second term is *δ*([*x*]) = ([*x*] − [*Ca*]*_in_*)/*τ*, where *τ* is the inverse of the pumping rate. It can be seen that the effect of coupling the Ca^2+^ concentration dynamics to the rest of the system is negligible if we use the same values for the parameters as in [31] and baseline intracellular Ca^2+^ concentration [*Ca*]*_in_* = 10^−4^. However, if the external Ca^2+^ concentration [*Ca*]*_out_* changes in Equation (7), as we propose to simulate here, [*Ca*]*_in_* and the model state can change, as well.

## 3. Results and Discussion

### 3.1. Microfluidic Simulations

**Figure 5:**
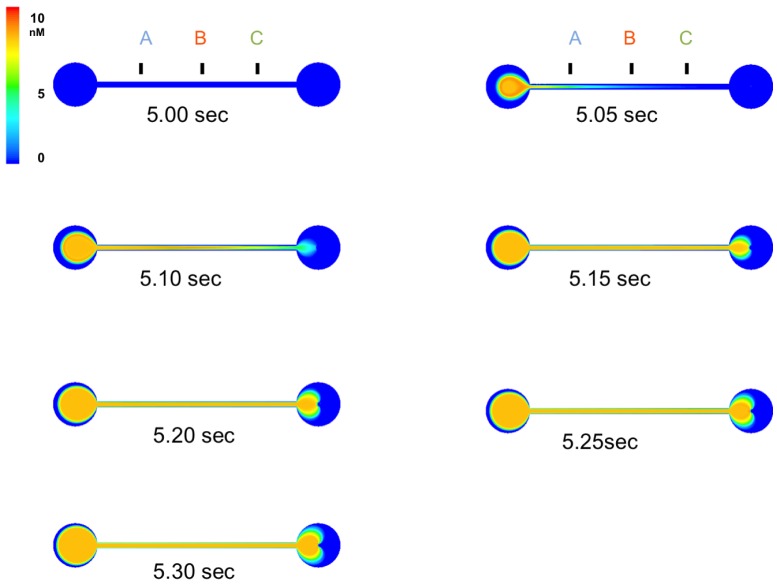
Computer simulation of predicted Ca^2+^ ion concentration change for flow through the whole device. The initial simulation starts at 5 s. The target concentration distributes quickly through the device. Details for specific points in one of the cases are presented in Figure 6.

**Figure 6:**
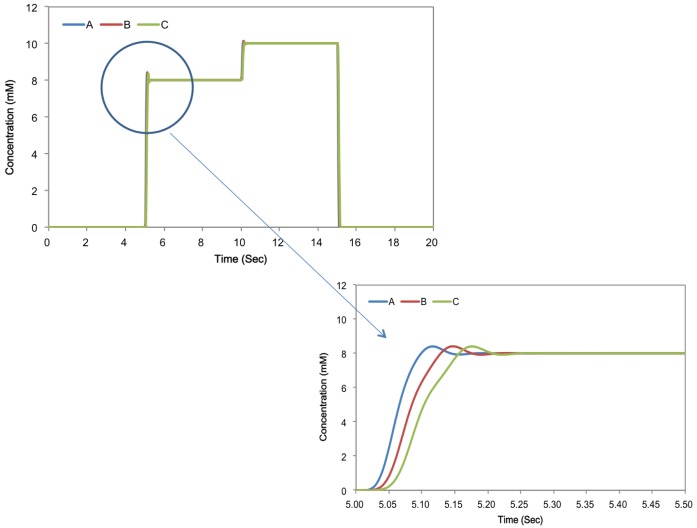
Predicted Ca^2+^ ion concentration change in the target Points A, B and C along the channel for Case 1. The top panel shows that the concentration distribution is essentially uniform at the relevant time scales (s). The bottom panel provides the predictions on a finer temporal scale, in which smoothing of the intended step function and the advection delay along the channel can be observed and estimated.

Figure 5 shows simulations of the flow dynamics within the device, after injection of a target concentration profile from Case 1 starting at 5.00 s. Subsequent frames track the distribution of increased concentration throughout the device. Figure 6 shows the concentration of Ca^2+^ ion at Points A, B and C, detailed in Figure 3. In Case 1, it took 5.10 s to achieve the initial maximum concentration at A, 5.14 s at B and 5.18 s at C, as expected from constant laminar flow. In Case 2, it took 8.93 s to achieve the maximum concentration at A, 8.97 s at B and 9.01 s at C. In this case, the trend was almost the same as Case 1; however, the concentration at A showed exactly the same shift from C, because the flow rate was fixed. This will be good for more precise control of the gradient and demonstrates the needed precision for flow rate control. The result from all test cases showed it was possible to control the concentration gradient at specific points by varying the flow.

### 3.2. Neural Simulations

We used the simulated Ca^2+^ concentration above as input to the neural models outlined in the Experimental Section. Our goal was to test whether changes in the Ca^2+^ concentration, like those triggered by signaling of other neurons or glial cells, could lead to changes in the activity of the modeled neuron. We show the results of these simulations as applied to the concentration signals in Figure 6 at Point A.

**Figure 7:**
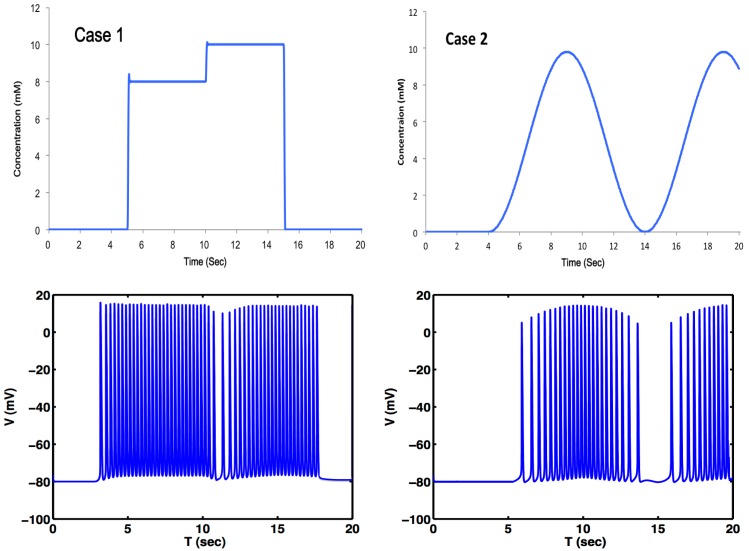
Simulations of a neural response to changing Ca^2+^ concentrations. Case 1: simulated Ca^2+^ concentration profile at Point A (top) and simulated neural response of a Hodgkin–Huxley-type model (Equation (4)) with Ca^2+^ channels (Equation (5)) at that point (**bottom**); Case 2: simulated Ca^2+^ concentration profile at Point A of the dynamically-changing concentration (**top**) and simulated neural response of a Hodgkin–Huxley-type model (Equation (4)) with Ca^2+^ channels (Equation (5)) at that point (**bottom**).

The bursting activity seen in Figure 7 is typically associated with changes to the neuron's membrane potential. The simulations in Figure 7 demonstrate that neural activity can be not only modulated, but also triggered by extracellular chemical concentrations.

### 3.3. Microfluidic Experiments

Testing solutions with different Ca^2+^ concentrations are obtained by mixing CaCl_2_ and deionized water in different volume ratios. During our experiment, two different solutions with concentrations of 8 mM and 10 mM are used. In order to monitor the real-time nanosensor's resistance caused by the different ionic solutions, a continuous flow of these aqueous solutions is injected into the micro-channel by a high-precision programmable syringe pump. To stimulate Case 1, an air bubble is introduced between these two solutions in the flow-feeding tube in order to eliminate unwanted mixing.

For generating dynamic flow, like that in Case 2, we use coupled syringe pumps. Two syringe pumps were programmed to work in synchronization, such that the analyte concentration can be varied at a constant total flow-rate. For this to occur, the individual pumps are operated with a phase between them. The sum of the flow-rate of the two fluid streams is a constant at any given instance of time. More complicated signals with various analytes (neurotransmitters, drugs and ions) can be generated by programming the pumps accordingly for a controlled neuronal stimulation.

**Figure 8:**
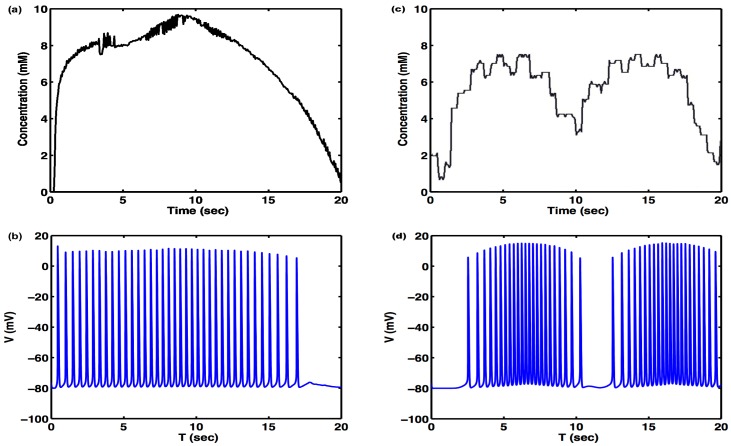
Measured Ca^2+^ concentration in a microfluidic device used to control a neural simulation. (**a**) Measured Ca^2+^ concentration profile obtained in the microfluidic experiments, with a profile approximating Case 1; (**b**) membrane voltage of a Ca^2+^-sensitive neuron using the experimentally-measured concentration (a) as an input; (**c**) measured Ca^2+^ concentration profile obtained in the microfluidic experiments, with a profile approximating Case 2; (**d**) membrane voltage of a Ca^2+^-sensitive neuron using the experimentally-measured concentration (c) as an input. Like the cases in Figure 7, the external Ca^2+^ concentrations here also trigger spike generation.

A semiconductor device analyzer is connected to the nanosensor on the microfluidic device for the real-time resistance measurement. In this experiment, the flow rate is precisely controlled at 0.3 mL/min, and the current-voltage (I-V) characteristics of the SWCNT sensors are obtained with the semiconductor device analyzer. Under the steady flow condition, the resistance of the SWCNT nanosensor increases as the Ca^2+^ concentration increases. The resulting concentration profile was effectively equivalent to Case 1 in Figure 7, except for the smaller scale and added small step between 8 and 10 mM concentrations. The resultant concentration profile is shown in Figure 8a. The outcome of the dynamic simulation, with a sinusoidal driving signal similar to Case 2 in Figure 7, is shown in Figure 8c.

### 3.4. Neural Simulations

The experimentally-induced and measured Ca^2+^ concentration, shown in Figure 8a, was used as an input for the external Ca^2+^ concentration of Equation (5). The resulting simulated response is shown in (b) of the same figure. A neural response was observed similar to the firing pattern for Case 1 in Figure 7, as expected, due to the similarity between observed concentrations of Ca^2+^ and the simulated concentrations shown in Figure 7a. The experimentally-induced and measured sinusoidal Ca^2+^ concentration, shown in Figure 8c, was used as an input for the external Ca^2+^ concentration of Equation (5). The resulting simulated response is shown in ( d) of the same figure. A neural response was observed similar to the firing pattern for Case 2 of Figure 7, as expected, due to the similarity between observed concentrations of Ca^2+^ and the simulated concentrations shown in Figure 7a.

### 3.5. Discussion

The chemical dynamic clamp is a promising new technology that will extend the power of the dynamic clamp method to the realm of neurochemical and neuromodulatory control and analysis of neural function. It will also provide an alternative method for the study of interactions between neurons and other tissues, which occur predominantly through chemical channels. The device and methods developed here take the initial steps in that direction.

The main impetus behind this work was our desire, as part of a bigger team, to understand the role of glia in the functioning of biological neural networks, specifically in the process of sleep [33,34]. Since glia interact with neurons and one another predominantly chemically, we are striving to build a tool that will allow us to manipulate chemical concentrations based on the function of glial and neural networks, as well as models of those functions with which to test our theories of neuro-glial interactions. The ultimate goal of this project will be to, for example, model a glial network and then instantiate the chemical outputs of such a network with the chemical dynamic clamp device proposed here, in order to test hypothesized effects of glia on neural cells. This experiment will be contrasted with the dynamics of a complete neuro-glial network, in which neurons and glia interact naturally.

In this manuscript, we demonstrate the benefits and drawbacks of using a microfluidic lab-on-a-chip as the mechanism through which a chemical dynamic clamp can be modulated. We simulated chip geometries and designs and their effects on Ca^2+^ flow inside the chip chambers. We show that the lab can maintain essentially a uniform concentration in the working volume; other approaches, like creating a concentration gradient, are also possible.

The design that we investigated showed the importance of mixing on the functionality of the chip [35]. The simulations used an effective mixer [36]; however, good mixing was achieved only after a prohibitively long mixer structure (and corresponding reaction delay) was introduced. Thus, for the actual experiments, we used a simplified microfluidic lab with specific concentrations prepared in advance. The situation can be remedied through the use of a bubble mixer [37]; however, that is too complex to simulate at the moment.

The work demonstrated the need for improved sensors with which to register the concentration of the controlled substance throughout the microfluidic chip. Since the outputs of these sensors are used as inputs to simulations and model-based microfluidic control, it is of paramount importance to have fast, reliable ways of registering these quantities. The current implementation of carbon nanosensors [24] was insufficient for the task, since it was too slow and noisy. We are preparing improvements to those nanosensors and investigating the use of well-established molecular probes [38] as an alternative sensing mechanism. Another consideration for integrated nanosensors is that they need to provide sufficient selectivity in addition to high sensitivity. Testing samples in a fluidic microenvironment contain multiple types of ions and electroactive entities. It is critical for the nanosensors to accurately identify and quantify each substance from a complex mixture. The simultaneous detection of multiple entities by sensors has been investigated previously using different approaches [39–41]. Among them, multiplexed electrochemical sensing can be used in our SWCNT nanosensors [42,43]. Functionalization by specific enzymes or receptors on the nanotube surface can enable high selectivity for each nanosensor. An array of specifically functionalized nanosensors can provide multiplexed sensing abilities, ideal for neurochemical applications.

The neural simulations with either simulated Ca^2+^ concentration (Figure 7) or measured Ca^2+^ concentration (Figure 8) demonstrate that neural activity can not only be modulated, but also triggered by extracellular chemical dynamics, opening new venues of research in the functional properties and information transmission of single neurons and biological neural networks.
